# Genotypic characterisation and antimicrobial resistance of *Pseudomonas aeruginosa* strains isolated from patients of different hospitals and medical centres in Poland

**DOI:** 10.1186/s12879-020-05404-w

**Published:** 2020-09-22

**Authors:** Marcin Brzozowski, Żaneta Krukowska, Katarzyna Galant, Joanna Jursa-Kulesza, Danuta Kosik-Bogacka

**Affiliations:** 1 Department of Medical Microbiology, Chair of Microbiology, Immunology and Laboratory Medicine, Pomeranian Medical University in Szczecin, Powstanców Wielkopolskich 72, 70-111 Szczecin, Poland; 2 Department of Laboratory Medicine; Chair of Microbiology, Immunology and Laboratory Medicine, Pomeranian Medical University in Szczecin, Powstanców Wielkopolskich 72, 70-111 Szczecin, Poland; 3 Independent of Pharmaceutical Botany, Pomeranian Medical University in Szczecin, Powstanców Wielkopolskich 72, 70-111 Szczecin, Poland

**Keywords:** *Pseudomonas aeruginosa*, Genotyping, Antimicrobial resistance, PFGE, Population structure

## Abstract

**Background:**

*Pseudomonas aeruginosa* is a Gram-negative bacteria responsible for infections in immunocompromised patients and is one of the most common causes of nosocomial infections particularly in intensive care and burn units. We aimed to investigate the population structure of *P. aeruginosa* strains isolated from patients at different hospital wards. Methods: We analysed the possible presence of *P. aeruginosa* epidemic or endemic strains in hospitals of the selected region. A genotyping analysis was performed for *P. aeruginosa* isolates (*n* = 202) collected from patients of eleven hospitals in north-western Poland. Collections of *P. aeruginosa* were genotyped using pulsed-field gel electrophoresis (PFGE). Phenotypic screening for antibiotic susceptibility was performed for the common antimicrobial agents.

**Results:**

*Pseudomonas aeruginosa* isolates were distributed among 116 different pulsotype groups. We identified 30 groups of clonally related strains, each containing from 2 to 17 isolates and typed the obtained 13 unique patterns, designated as A, D, E, J, K, M, N, Ó, P, T, X, AC, AD, and AH. The two largest clusters, D and E, contained 17 and 13 isolates, respectively. Strains of these groups were continuously isolated from patients at intensive care units and burn units, indicating transmission of these strains.

**Conclusions:**

In this study, we demonstrate the clonal relatedness of *P. aeruginosa* strains and their constant exchange in hospitals over a period of 15 months. The obtained results indicate a predominantly non-clonal structure of *P. aeruginosa*.

## Background

*Pseudomonas aeruginosa* is a Gram-negative opportunistic pathogen that causes nosocomial infections for patients with pre-existing lung disease, including cystic fibrosis or chronic obstructive pulmonary disease, or patients on mechanical ventilation in the intensive care (ICUs). Eradication of *P. aeruginosa* in hospitals is especially problematic due to its intrinsic resistance to many antibiotic classes and its capacity to acquire resistance to all effective antibiotics [1, 2]. *Pseudomonas aeruginosa* colonization of patients may originate from different exogenous sources such as sanitary installations (sinks, hot tubs, showers, etc.), contaminated diagnostic devices, mechanical ventilation, and cleaning equipment [3]. Infections of healthy individuals are rather rare; however, healthy colonised patients can serve as a continuous source of *P. aeruginosa* transmission [4].

*Pseudomonas aeruginosa* outbreaks occur in ICUs, neonatal ICUs, burn units (BU), haematological units, and other hospital wards where immunocompromised or critically ill patients are treated [5–8]. ICU patients are particularly at risk of *P. aeruginosa* infections due to the length of their stay in the medical ward, the severity of their illness and exposure to invasive medical procedures. It has been reported that *P. aeruginosa* infections of ICU patients primarily manifest as acute lung infections [6]. According to data from the European Centre for Disease Prevention and Control (ECDC), *P. aeruginosa* is the most common cause of respiratory pneumonia and the third most frequent agent causing urinary tract infections at European ICUs [9]. Among the distinguished causes of *P. aeruginosa* infections, the authors highlight the main role of inappropriate disinfection rather than resistance to the disinfectant used [10, 11].

According to previous reports, *P. aeruginosa* has a non-clonal population structure [12]. It has been established that the diversity of *P. aeruginosa* clones in extant populations is mostly generated by a frequent recombination of strains [12, 13]. This mechanism of undergoing recombination also significantly contributes to the continuous evolution of *P. aeruginosa* within the lungs of cystic fibrosis patients [14]. Despite the non-clonal population, it is possible to distinguish endemic, epidemic or even pandemic *P. aeruginosa* strains disseminating in hospitals [12, 15, 16]. Despite the intrinsic resistance to several antimicrobials, *P. aeruginosa* may acquire additional resistance mechanisms to all routinely used antipseudomonal drugs [1]. Multidrug resistance to antimicrobial agents of strains responsible for nosocomial outbreaks have been frequently noted by the authors in previous investigations [17–19]. The increased antimicrobial resistance of *P. aeruginosa* strains is contributing to a higher mortality rate of infected patients, longer hospitalization, more severe illness, and higher costs of treatment [1].

In this study, we describe the population structure and antimicrobial resistance rates of *P. aeruginosa* strains isolated from patients in different medical units of hospitals in Poland. The aim of this study was to establish the population structure, presence and distribution of possible epidemic and endemic *P. aeruginosa* strains among patients of hospitals. We analysed antimicrobial resistance rates of selected strains. The results provided by this study did not reveal the presence of an outbreak, although some strains were acknowledged as endemic due to their prolonged isolation in hospitals.

## Methods

### Bacterial isolates

*Pseudomonas aeruginosa* isolates (*n* = 742) were collected from 541 patients of 11 different hospitals and medical centres located in north-western Poland between December 2015 and March 2017. From the first isolate per patient, 202 were randomly selected for typing by Pulsed Field Gel Electrophoresis and antimicrobial drug resistance analysis.

Hospitals where the isolation was carried out were designated with letters H1 – H11. The largest number of selected isolates (*n* = 174) originated from four major general hospitals H1-H4, located in three different cities of north-western Poland. The isolation of *P. aeruginosa* strains was less frequent in other smaller specialised hospitals and care facilities in the chosen region of Poland and thus the number of strains from these medical units is lower. Detailed information regarding the hospitals and the amount of strains collected at different hospital wards is included in Table 1. The selected strains were isolated from blood (*n* = 35), the lower respiratory tract (*n* = 76), wounds (*n* = 65), urine (*n* = 12), and ear and eye swab samples (*n* = 14). Firstly, *P. aeruginosa* isolates were identified in the hospitals’ microbiology laboratories. Further genus identification was performed using the PCR method described by Spilker et al. [20].

**Table 1:** Sample collection locations and number of isolates collected from examined wards (ICU, intensive care unit; BU, burn unit; SU, surgical unit; OU, other units; n, number of isolates; H1, Multispeciality Voivodeship Hospital in Gorzów Wielkopolski; H2, Regional Specialist Hospital in Gryfice; H3, Independent Public Clinical Hospital no. 1, Pomeranian Medical University in Szczecin; H4, Independent Public Clinical Hospital no. 2, Pomeranian Medical University in Szczecin; H5, Specialised Zdroje Hospital in Szczecin; H6, Sokolowski Specialist Hospital in Szczecin; H7, Hospital of the Ministry of the Interior and Administration in Szczecin; H8, West Pomeranian Oncology Centre in Szczecin; H9, West Pomeranian Hospice for Children in Szczecin; H10, Independent Public Clinical Hospital no. 1, Pomeranian Medical University in Police; H11, Residential Health Care Centre in Resko)

Hospital	City	Hospital type	Number of isolates collected from medical units of different types				
			ICU	BU	SU	OU	All units
H1	Gorzów Wlkp.	General hospital	9	0	15	15	39
H2	Gryfice	General hospital	18	32	8	5	63
H3	Szczecin	General hospital	35	0	2	11	48
H4	Szczecin	General hospital	16	0	4	4	24
H5	Szczecin	Specialised hospital	1	3	0	2	6
H6	Szczecin	Specialised hospital	0	0	2	4	6
H7	Szczecin	Specialised hospital	1	0	2	2	5
H8	Szczecin	Clinic	0	0	1	1	2
H9	Szczecin	Care facility	0	0	0	1	1
H10	Police	Branch of hospital H3	1	0	1	4	6
H11	Resko	Care facility	0	0	0	2	2

### Antimicrobial susceptibility testing

The antipseudomonal drugs assessed in the study included carbapenems (meropenem, imipenem), cephalosporins (ceftazidime, cefepime), aminoglycosides gentamicin, amikacin, tobramycin, fluoroquinolone (ciprofloxacin), a penicillin + β-lactamase inhibitor (piperacillin-tazobactam) and polymyxin (colistin). To identify antimicrobial susceptibility, the disk diffusion method was performed. *Pseudomonas aeruginosa* ATCC 27853 was used as quality control. Minimal Inhibitory Concentration (MIC) of colistin was determined with a broth microdilution-based method (ComASP™ Colistin test, Liofilchem) as the disk diffusion method is no longer recommended to evaluate polymyxins susceptibility. Concentrations used varied between 0.25 μg/ml and 16 μg/ml. Tests were performed and interpreted according to the EUCAST guidelines [21].

### DNA isolation

The chromosomal *P. aeruginosa* DNA was isolated with the CHEF Bacterial Genomic DNA Plug Kit (Bio-Rad Laboratories) with some modifications of the manufacturer’s instructions. *Pseudomonas aeruginosa* isolates were cultured overnight at 37 °C on cetrimide agar. In the first stage of isolation, bacterial suspensions in PBS equivalent to 4–4.5 McFarland were prepared, then incubated in a water bath at 37 °C for 20 min. A 100 μl sample of each bacterial suspension was mixed with 100 μl of melted 2% agarose gel at 50 °C. These mixtures were allowed to solidify in plug moulds. The resulting agarose plugs were mixed by inversion with a proteinase buffer with 20 μl of proteinase (CHEF Bacterial Genomic DNA Plug Kit, Bio-Rad Laboratories), then incubated for 20 h at 50 °C. After incubation, the proteinase solution was removed and the plugs washed three times with 1 ml of 1x wash buffer from the commercial kit (1 wash/1 h). The plugs were either stored at 4 °C or used immediately in further analyses.

### Restriction endonuclease digestion

Before restriction digestion, plugs were washed once in a 0.1x wash buffer for 1 h, followed by a subsequent wash with a tango buffer (Thermofisher), again for 1 h. For the restriction endonuclease digestion, a *SpeI* restriction enzyme was used (Thermofisher). Each plug was resuspended in fresh tango buffer and 50 U of *SpeI* was added, followed by incubation for 20 h at 37 °C.

### PFGE and data analysis

One third of each plug was loaded into a 1.2% agarose, 0.5% TBE gel. Pulsed-field gel electrophoresis (PFGE) was conducted with the CHEF-DRIII system (Bio-Rad Laboratories, USA), using 0.5x TBE as a running buffer and the following run conditions: 6 V/cm, 14 °C, for 20 h with initial and final switch times 5 and 45 s, respectively. *Pseudomonas aeruginosa* ATCC 27853 was applied as a positive control. The gel was stained with ethidium bromide to visualise DNA bands. Obtained images were exported to BioNumerics software ver. 7.6. (Applied Maths). PFGE patterns were compared using the Dice similarity coefficient. A dendrogram showing genetic relatedness of the strains was constructed by the single linkage method with 1% tolerance and 1% optimisation of band position. PFGE fingerprints with ≥85% similarity were categorised as clonally related.

## Results

### Population of *P. aeruginosa* strains

**Fig. 1:**
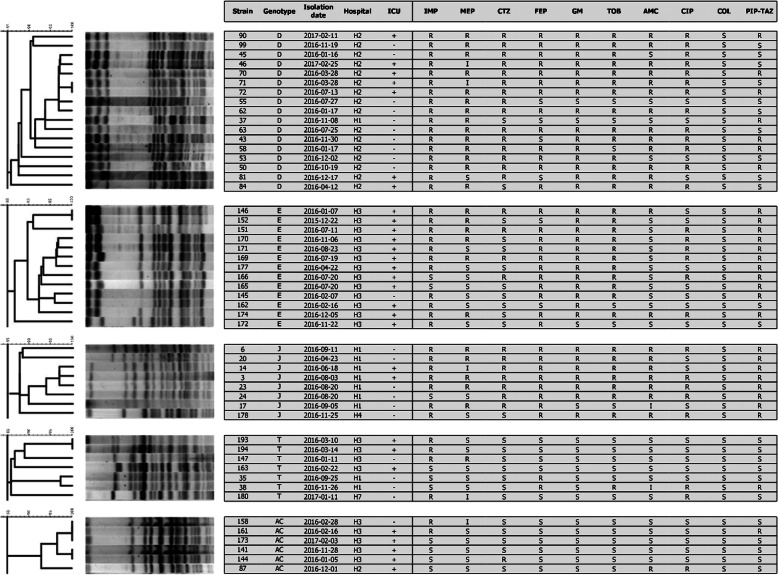
Restriction patterns and characteristics of isolates belonging to PFGE types containing five or more strains. Strains isolated from ICUs are marked with + (ICU, intensive care unit, IMP, imipenem; MEP, meropenem; CTZ, ceftazidime; FEP; cefepime, GM, gentamycin; TOB, tobramycin; AMC, amikacin; CIP, ciprofloxacin; PIP-TAZ, piperacillin-tazobactam)

A clonal analysis of *P. aeruginosa* strains was carried out for 202 strains isolated from 11 different hospitals located in north-western Poland. *Pseudomonas aeruginosa* isolates were distributed among 116 different pulsotype groups. Thirty clusters consisted of two or more strains. The clusters were assigned with different letter designations from A to AH. A total of 86 clusters contained only one strain. Strains clonally related with at least one other isolate in this study were found in all hospitals except hospital H6. Five groups of clonally related strains (D, E, J, T, AC) included more than 5 strains (Fig. 1). The most prevalent D cluster included 17 strains isolated from two hospitals H2 (*n* = 16) and H1 (*n* = 1). Strains of those groups were isolated at the ICU (*n* = 7), BU (*n* = 8), and surgical unit (SU, *n* = 1), from December 2015 to March 2017. All isolates of group D were resistant to imipenem. Group E included 13 isolates collected between December 2015 and December 2016. Isolates of this cluster were isolated from different patients of an ICU (*n* = 12) and orthopaedic unit (*n* = 1) of hospital H3. Isolates of group D and E represented 8.6 and 6.4% of all isolates collected, respectively. Strains of cluster E were isolated 12 times from patients at ICUs of hospital H3, which stands for 25% (12/48) of all isolates collected in this hospital. Strains of cluster D were collected 16 times from patients of a burn unit and the ICU of H2, representing 25.4% of all isolates from this medical centre. Most of the isolates of cluster D were resistant to carbapenems (14/17), aminoglycosides (12/17), and other antimicrobials tested except piperacillin with tazobactam and colistin. Similar high rates of resistance to various antimicrobial classes were noted for strains from pulsotype E. Both clusters D and E share resistance to imipenem and aminoglycosides.

Strains of pulsotypes J, T, and AC were isolated 8, 7 and 6 times from different patients. Restriction patterns, hospitals, wards, isolation dates, and antimicrobial susceptibility patterns of *P. aeruginosa* strains isolates belonging to the major pulsotype groups are shown in Fig. 1. In this study, we found 13 clusters of strains: A, D, J, K, M, N, Ó, P, T, X, AC, AD, and AH that were isolated from patients of two or more hospitals. Strains of group T were collected in 3 different hospitals H1 (*n* = 2), H3 (*n* = 4), and H7 (*n* = 1). Isolates of the T cluster were susceptible to most of the antimicrobials tested, except for imipenem, for which 4 strains were resistant.

**Fig. 2:**
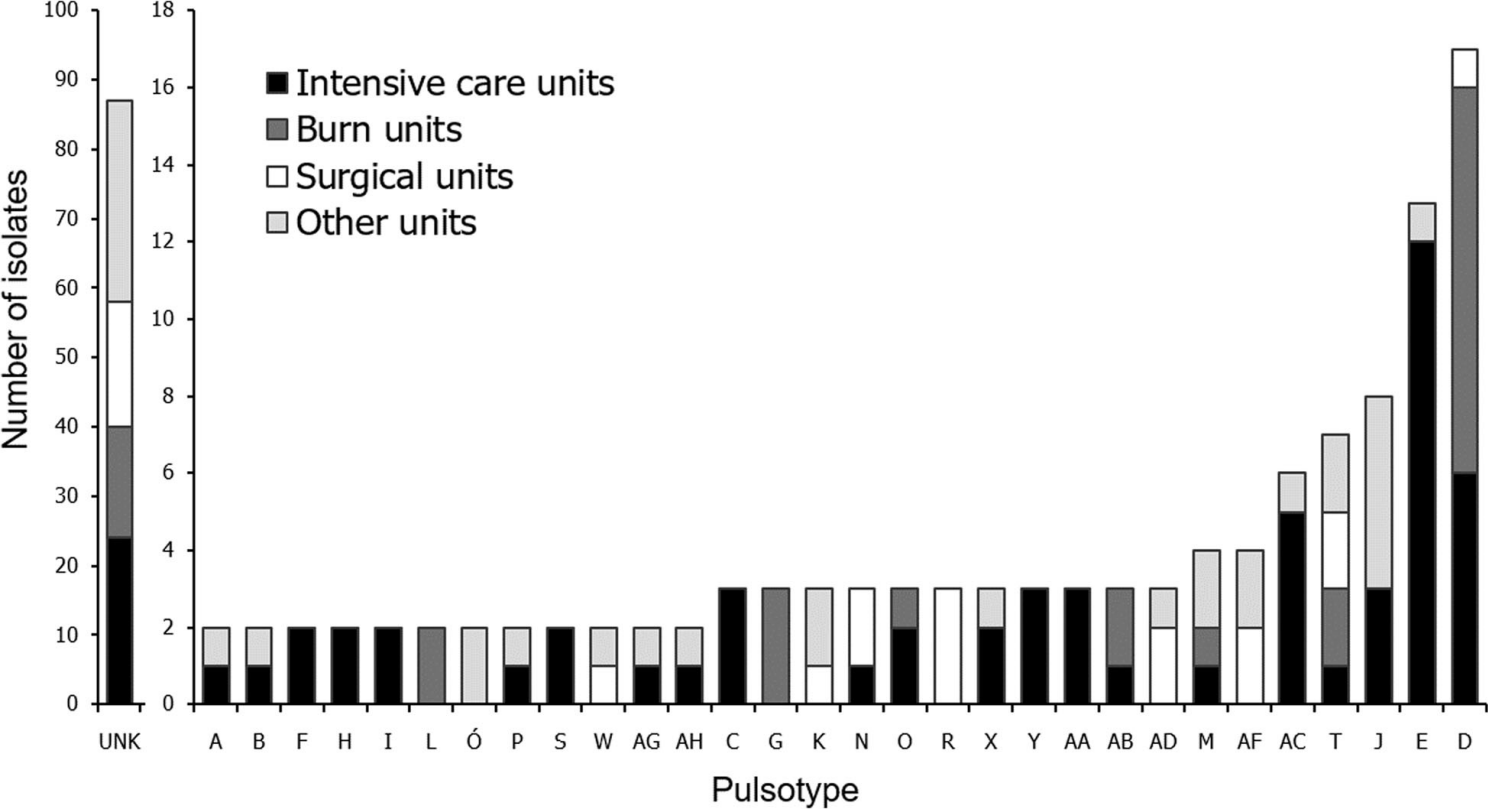
Distribution of isolates belonging to different PFGE types among intensive care units, burn units, surgical units, and other units (UNK, unique pulsotype)

Most of the *P. aeruginosa* strains from this study were isolated in ICUs (*n* = 81). All remaining isolates were isolated in BU (*n* = 35), SU (*n* = 35), and other units (*n* = 51). Strains from clusters containing two and more strains were most frequently isolated in ICUs. Among strains isolated from these units, 56/81 (69.1%) were clonally related to at least one other strain in this study. In BU, 21/35 (60%) isolates have similar restriction patterns shared by two or more strains. In Fig. 2, we demonstrated a distribution of PFGE clusters of various sizes among medical units of different types.

Overall, our findings show the presence of multiple *P. aeruginosa* pulsotypes and a constant exchange of strains inside a single hospital ward, between different hospital wards and between different hospitals. Part of the clonally related strains were constantly isolated from different patients of the same hospital ward. Results presented in this work suggest a non-clonal structure of *P. aeruginosa* population of strains isolated at hospital centres located in north-western Poland.

### Antimicrobial resistance prevalence

Resistance to tested antipseudomonal drugs was as follows (202 strains): imipenem 67.8% (*n* = 137), meropenem 29.2% (*n* = 59), ceftazidime 33.2% (*n* = 67), cefepime 42.6% (*n* = 86), piperacillin-tazobactam 39.6% (*n* = 80), gentamicin 37.6% (*n* = 76), amikacin 30.2% (*n* = 61), tobramycin 38.1% (*n* = 77), ciprofloxacin 39.6% (*n* = 80), and colistin 3.0% (*n* = 6). The prevalence of resistance to both carbapenems was 29.2%, and for all aminoglycosides 22.8%. Combined resistance to carbapenems and aminoglycosides was 14.4% (*n* = 29). Out of 202 isolates in this study, 48% (*n* = 97) were multidrug-resistant (MDR). Multidrug-resistance was defined as not susceptible to 3 or more antibiotic classes (*n* = 20) [22].

Among MDR strains, 71.1% (*n* = 69) of isolates were clonally related with at least one more isolate in the study. However non-MDR susceptible strains (*n* = 105) less frequently exhibited clonal relatedness with other strains 44.8% (*n* = 47).

We observed a variation in antimicrobial resistance rates between different hospital wards. Strains isolated from BU were most often resistant to the tested antimicrobials except piperacillin with tazobactam, ciprofloxacin and colistin. Data regarding antimicrobial resistance of strains isolated at different medical units are shown in Table 2.

**Table 2:** Antimicrobial resistance rates of strains isolated from patients of different medical wards (ICU, intensive care unit; BU, burn unit; SU, surgical unit; OU, other units; n, number of isolates; %R, resistance rate; 95% CI, 95% confidence interval)

Antimicrobial agent	ICU (*n* = 81)		BU (*n* = 35)		SU (*n* = 32)		OU (*n* = 54)		All units (*n* = 202)	
	%R	95% CI	%R	95% CI	%R	95% CI	R%	95% CI	R%	95% CI
Imipenem	71.1	9.6	72.7	15.2	58.3	19.7	65.5	12.6	67.8	6.4
Meropenem	28.9	9.6	51.5	17.1	20.8	16.2	21.8	10.9	29.2	6.3
Ceftazidim	32.5	9.9	51.5	17.1	37.5	19.4	23.6	11.2	33.2	6.5
Cefepime	43.4	10.5	48.5	17.1	41.7	19.7	38.2	12.8	42.6	6.8
Piperacillin/tazobactam	48.2	10.6	24.2	14.6	37.5	19.4	38.2	12.8	39.6	6.7
Gentamicin	33.7	10	57.6	16.9	33.3	18.9	34.5	12.6	37.6	6,7
Tobramycin	31.3	9.8	57.7	16.9	45.8	19.9	34.5	12.6	38.1	6.7
Amikacin	19.3	8.3	57.8	16.9	33.3	18.9	30.9	12.2	30.7	6.4
Ciprofloxacin	28.9	9.6	51.5	17.1	66.7	18.9	34.5	12.6	39.1	6.4
Colistin	1.2	2.3	0	–	8.3	11.0	5.5	6.0	3.0	2.4

## Discussion

This study allows for a description of the population structure of *P. aeruginosa* clinical isolates collected from hospitals of north-western Poland. It revealed the presence of 30 clusters of related strains and 86 unique strains. The obtained results indicate a predominantly non-clonal population structure and continuous exchange of *P. aeruginosa* strains between patients of the same and different hospital wards, and even between different hospitals in north-western Poland. Similar results have been presented in the previous epidemiological study conducted in this region [23]. Authors indicated a frequent transmission of *P. aeruginosa* strains between patients in medical centres. Our results are also in concordance with other epidemiological studies demonstrating a non-clonal population structure of *P. aeruginosa* [12, 15, 16]. It has been reported that the relatively high recombination frequency of *P. aeruginosa* strains is considered to be a key driver of the multiclonal population structure [12, 13].

Our results indicate continuous isolation of clonally related *P. aeruginosa* pathogens from different patients on the same ward. Hospital staff were not aware about the spread of pathogens during that time and did not report any outbreak. The two largest PFGE types, D and E, were described as endemic. Strains of those groups were isolated 17 and 13 times in hospital wards during a period of 1 year; however, no increase in infection rates was reported by the hospital staff. Isolates of cluster E were isolated 12 times from patients at ICUs of a hospital in Szczecin (H3), which stands for 25% of all isolates from that hospital. Strains of cluster D represented 25.4% of all isolates from the burn unit and ICUs of a hospital in Gryfice (H2) (Fig. 2). In other studies, the most prevalent PFGE types were shared by 22–52% of all *P. aeruginosa* isolates in a single hospital or medical unit [24–26]. Strains in these studies were continuously isolated from patients of hospital units and were described as endemic. Due to the hospital selection pressure, the resistance of endemic strains to multiple antimicrobial agents appears to be the determining factor of their endemicity [27].

Patients of intensive care and burn units are particularly at risk of nosocomial infections caused by *P. aeruginosa* [6, 18]. The results of this work demonstrate that the transmission of *P. aeruginosa* strains was most frequent in intensive care and burn units. Endemic strains of clusters D and E were also mostly isolated from patients of medical wards of these types.

In other medical units, the transmission of strains between patients was also possible, however, less frequent. The risk of colonisation by *P. aeruginosa* hospital strains at ICUs is higher presumably due to the prolonged stay of patients, the severity of their illness and exposure to invasive medical procedures [28]. Detection of strains disseminated in hospital wards seems critically important for immunocompromised patients and other patients susceptible to *P. aeruginosa* infections.

In this study clonally related strains were also isolated from patients of two or more hospitals, which indicates spread of these pathogens between medical centres in north-western Poland. It is thought that related strains can be transferred between different hospitals via hospital staff or their residents [29].

Epidemic and endemic strains are often multiple drug resistant which is responsible for the increased mortality of those patients. Inappropriate empirical therapy is perceived as the main factor contributing to increased mortality [30]. This study, in a similar way, demonstrates that MDR strains were more frequently found in clusters of clonally related strains. Among MDR *P. aeruginosa* strains, 71.1% exhibited clonal relatedness with at least one other strain. Strains of cluster D and E were resistant to imipenem and aminoglycosides. Resistance to other antimicrobials was also common in these clusters.

Detection and elimination of dissemination of high-risk clones in many cases is not possible with the use of simple epidemiological data alone [12, 31]. This is also the case in this study, where we demonstrated the presence of endemic strains at ICU and burn units that were not detected by hospital staff. Epidemiological studies with the use of molecular methods were not conducted at these wards at the time when clonally related strains were isolated. Thus, the use of molecular typing methods is necessary, especially at various ICU and BU, to establish a possible transmission of clonally related strains between patients that may appear in the future. The establishment of new infection prevention and control strategies should also be considered.

The obtained data regarding antimicrobial resistance indicate that levels of resistance of *P. aeruginosa* strains isolated from north-western Poland are comparable with results for the whole country [32]. According to a 2017 ECDC survey, the average resistance rate to carbapenems (imipenem+meropenem) was 24.2% across Poland, whereas in this research, 28.7% of strains were resistant to carbapenems. Discrepancies between results can be due to different infection control management in hospitals, misuse of antimicrobial agents, sanitation and distributions of strains in the region [33].

The lowest resistance rate among used aminoglycosides has been observed for amikacin. Relatively lower amikacin resistance rates of *P. aeruginosa* strains were also noted in other studies. Sader et al. [34] compared resistance rates of *P. aeruginosa* strains originating from multiple medical centres in the USA, and recorded susceptibility rates to gentamicin*,* tobramycin, and amikacin of 88, 90, and 98%, respectively. Similar data was obtained from research conducted in China, where amikacin was the second (after colistin) most effective antibiotic [35]. Lower amikacin resistance rates comparing to resistance to other aminoglycosides was also observed for multi-drug resistant *P. aeruginosa* strains [36]. Results presented in this and other epidemiological studies suggest that the development of amikacin resistance is less common than for other aminoglycosides in *P. aeruginosa* strains, and therefore that the use of amikacin could provide a better chance of success in empiric therapy. The lowest resistance rate indicated was that for colistin (~ 3%). Previously reported colistin resistance rates among various *P. aeruginosa* strains worldwide varied between 0 to 36% [37, 38]. Colistin is used to treat *P. aeruginosa* infection due to the MDR profiles of many strains, which mean alternative antibiotics cannot be prescribed. However, colistin is not used routinely due to its diverse side effects, including neuro- and nephrotoxicity [39].

This work has certain limitations. We acknowledge that the collected strains represented only a part of *P. aeruginosa* strains isolated from patients at hospitals in north-western Poland. We cannot assure that some unique strains were not in fact clonally related with other strains from other medical centres. However, this is the largest *P. aeruginosa* genotyping study that was ever performed in the region of north-western Poland. The investigated amount of strains allowed us to demonstrate a non-clonal population structure and reveal the presence of endemic *P. aeruginosa* strains in hospitals of the selected region. Secondly, this analysis relied on clinical *P. aeruginosa* strains collected only from patients. Environmental samples were not collected for this study. Additional strains from nosocomial environments could help us determine the possible hospital sources of *P. aeruginosa* strains in hospitals. Furthermore, it would be interesting to look into the mechanisms of resistance of multidrug-resistant strains, especially clonally related strains that were spreading over the ICU and BU. Metallo-β-lactamase (MBL) producers are commonly found in hospitals of Western-Europe, and are frequently responsible for outbreaks in ICU units [40–42]. Therefore it would be interesting to know the proportion of MBL producers in our hospitals.

The use of PFGE method for genotyping could also be considered as a drawback of this study. It is time-consuming, relatively expensive, and the results are difficult to compare interlaboratory [43]. Additional implementation of other typing methods would have additional value to the study. Performing MLST would allow us to compare results internationally.

However, there are a number of genotyping studies in which PFGE is still used [44, 45]. Although this method was first introduced in 1986, it is still frequently used for typing clinically relevant microorganisms.

## Conclusions

The results of this study show a constant exchange of different *P. aeruginosa* isolates between patients of medical wards in the same wards, different wards and even different hospitals of Poland. A high number of different PFGE types indicates a non-clonal population structure of *P. aeruginosa* species. These results are in congruence with previous similar studies. The results indicate the absence of a *P. aeruginosa* outbreak in Poland, although some endemic strains are undoubtedly present in intensive care and burn units. An antimicrobial resistance analysis showed that the antimicrobial resistance rates of *P. aeruginosa* isolates from hospitals of the selected region are comparable with the results from other parts of Poland. The revealed prolonged isolation of highly resistant endemic strains in hospitals indicates that more frequent use of genotyping methods in hospital wards, especially in intensive care and burn units, should be considered in the future.

## Data Availability

The data sets used and/or analyzed during the current study are available from the corresponding author on reasonable request.

## Abbreviations

ICU: Intensive care unit
BU: Burn unit
SU: Surgical unit
OU: Other units
n: Number of isolates
H1: Multispeciality Voivodeship Hospital in Gorzów Wielkopolski
H2: Regional Specialist Hospital in Gryfice
H3: Independent Public Clinical Hospital no. 1, Pomeranian Medical University in Szczecin
H4: Independent Public Clinical Hospital no. 2, Pomeranian Medical University in Szczecin
H5: Specialised Zdroje Hospital in Szczecin
H6: Sokolowski Specialist Hospital
H7: Hospital of the Ministry of the Interior and Administration in Szczecin
H8: West Pomeranian Oncology Centre in Szczecin
H9: West Pomeranian Hospice for Children in Szczecin
H10: Independent Public Clinical Hospital no. 1, Pomeranian Medical University
H11: Residential Health Care Centre in Resko
